# Supplementary material for: Frequent Germline and Somatic Single Nucleotide Variants in the Promoter Region of the Ribosomal RNA Gene in Japanese Lung Adenocarcinoma Patients
Source: Cells. 2020 Nov 3;9(11):2409. doi: 10.3390/cells9112409 (PMC7692307; doi:10.3390/cells9112409)
Supplement: Supplementary file 1 [file cells-09-02409-s001.zip › Supplementary files/cells-942745 - Supplementary Figures.pdf]

Figure S1

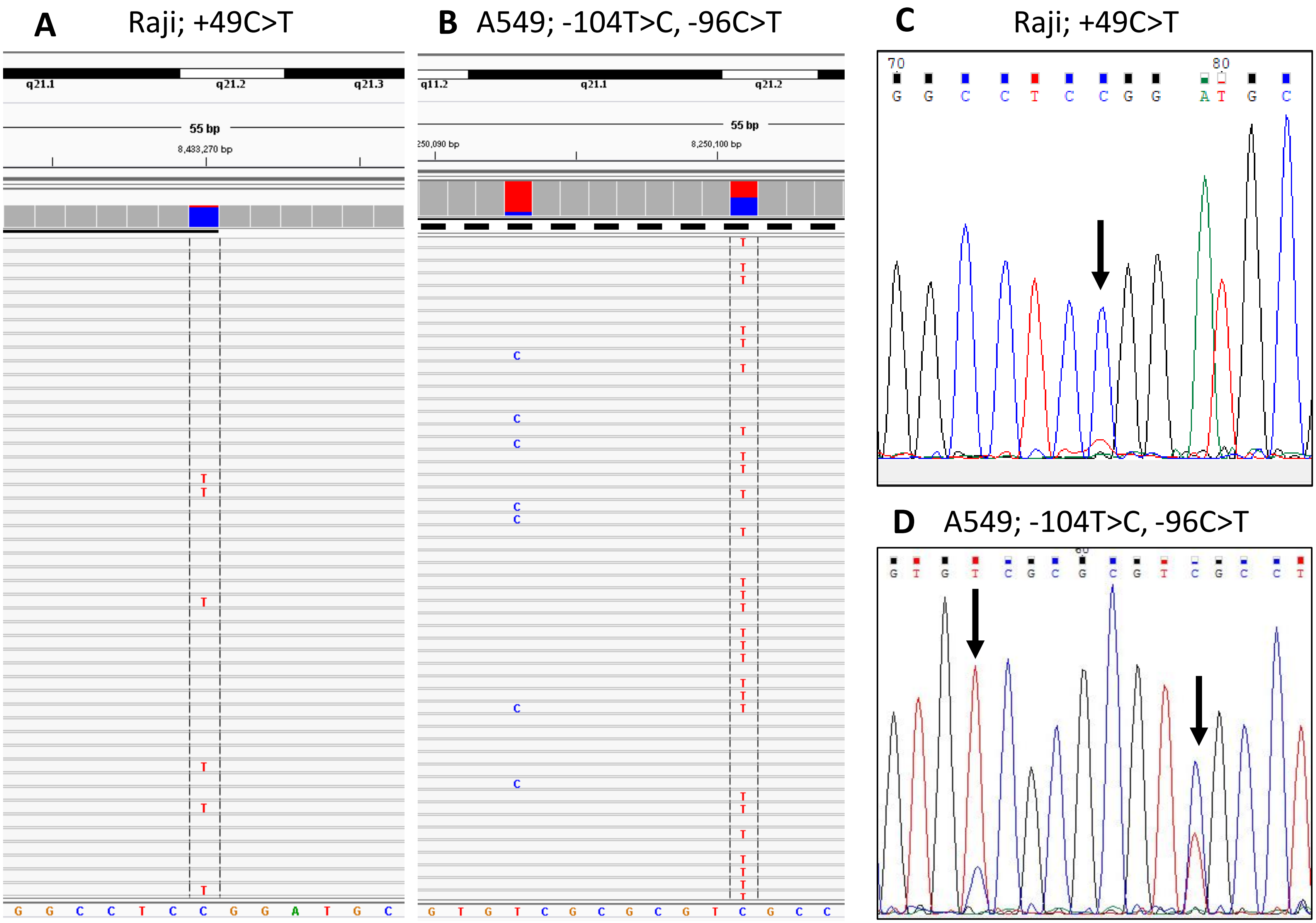

A
Raji; +49C>T
B
A549; -104T>C, -96C>T
C
Raji; +49C>T
D
A549; -104T>C, -96C>T
q21.1
q21.2
q21.3
55 bp
8,433,270 bp
q11.2
250,090 bp
8,250,100 bp

Figure S2

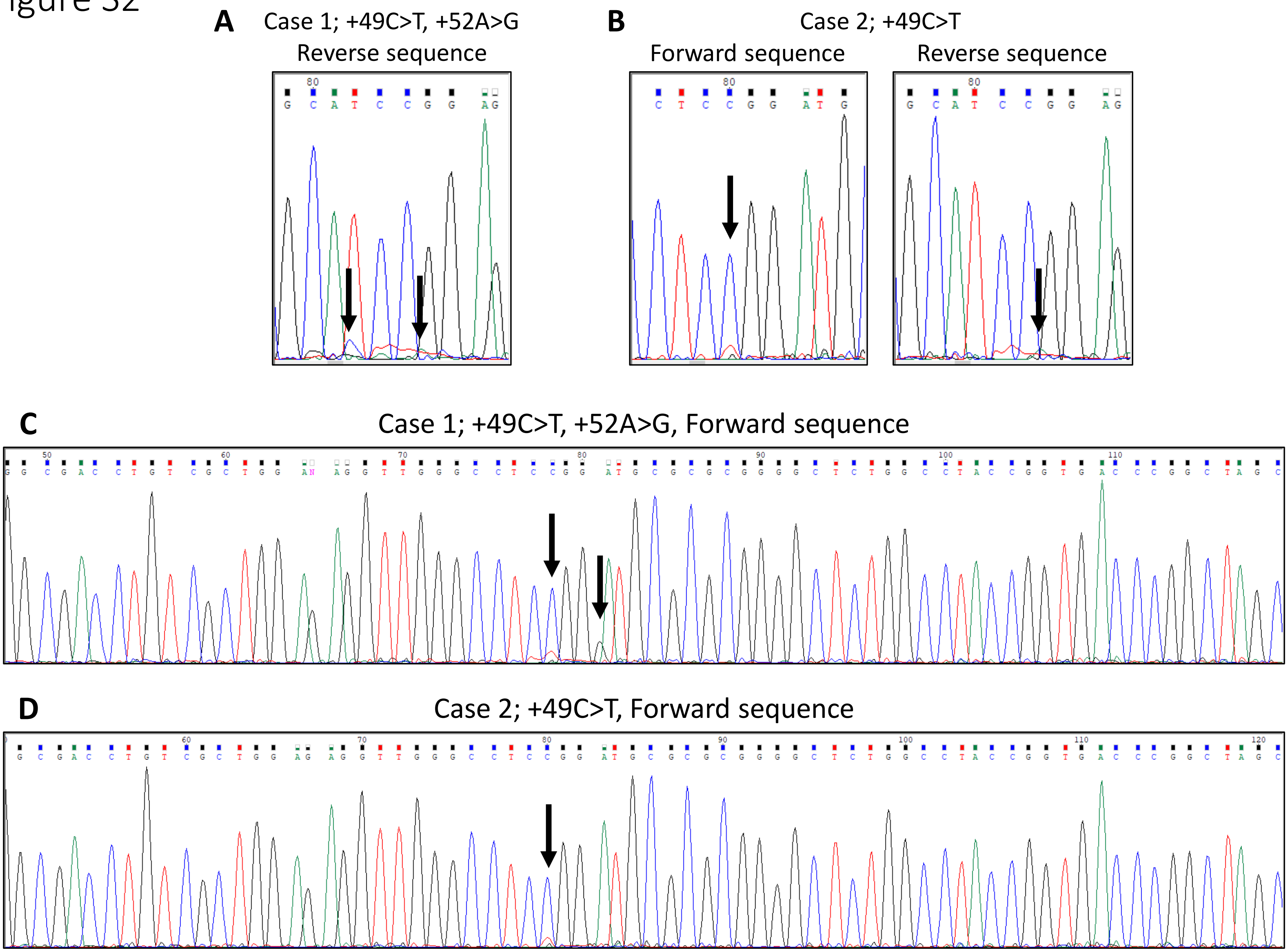
A
Case 1; +49C>T, +52A>G
Reverse sequence
B
Case 2; +49C>T
Forward sequence
Reverse sequence
C
Case 1; +49C>T, +52A>G, Forward sequence
D
Case 2; +49C>T, Forward sequence

Figure S3

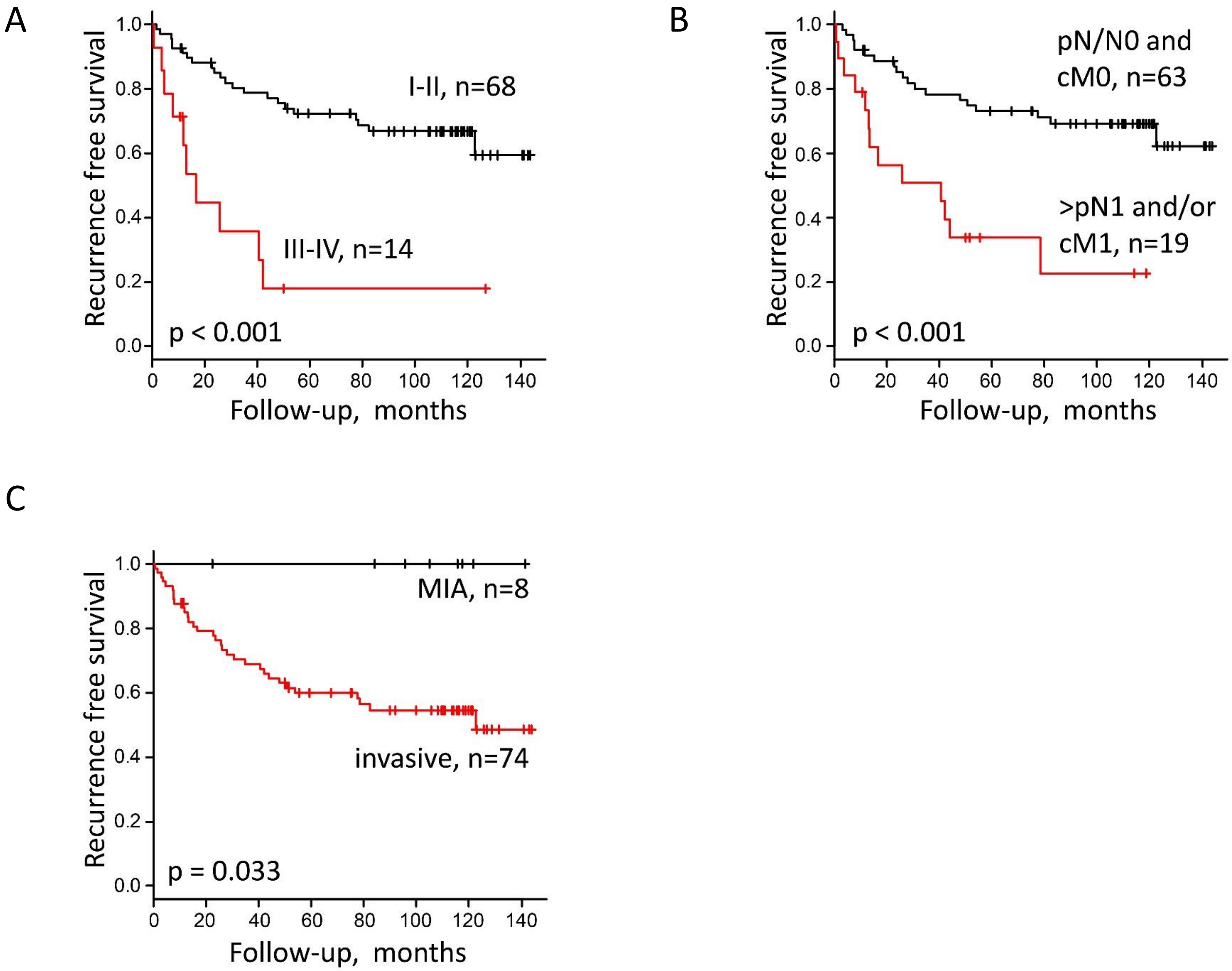
A
Recurrence free survival
1.0
0.8
0.6
0.4
0.2
0.0
I-II, n=68
III-IV, n=14
p < 0.001
0
20
40
60
80
100
120
140
Follow-up, months
B
Recurrence free survival
pN/N0 and
cM0, n=63
>pN1 and/or
cM1, n=19
p < 0.001
Follow-up, months
C
Recurrence free survival
MIA, n=8
invasive, n=74
p = 0.033
Follow-up, months

Figure S4

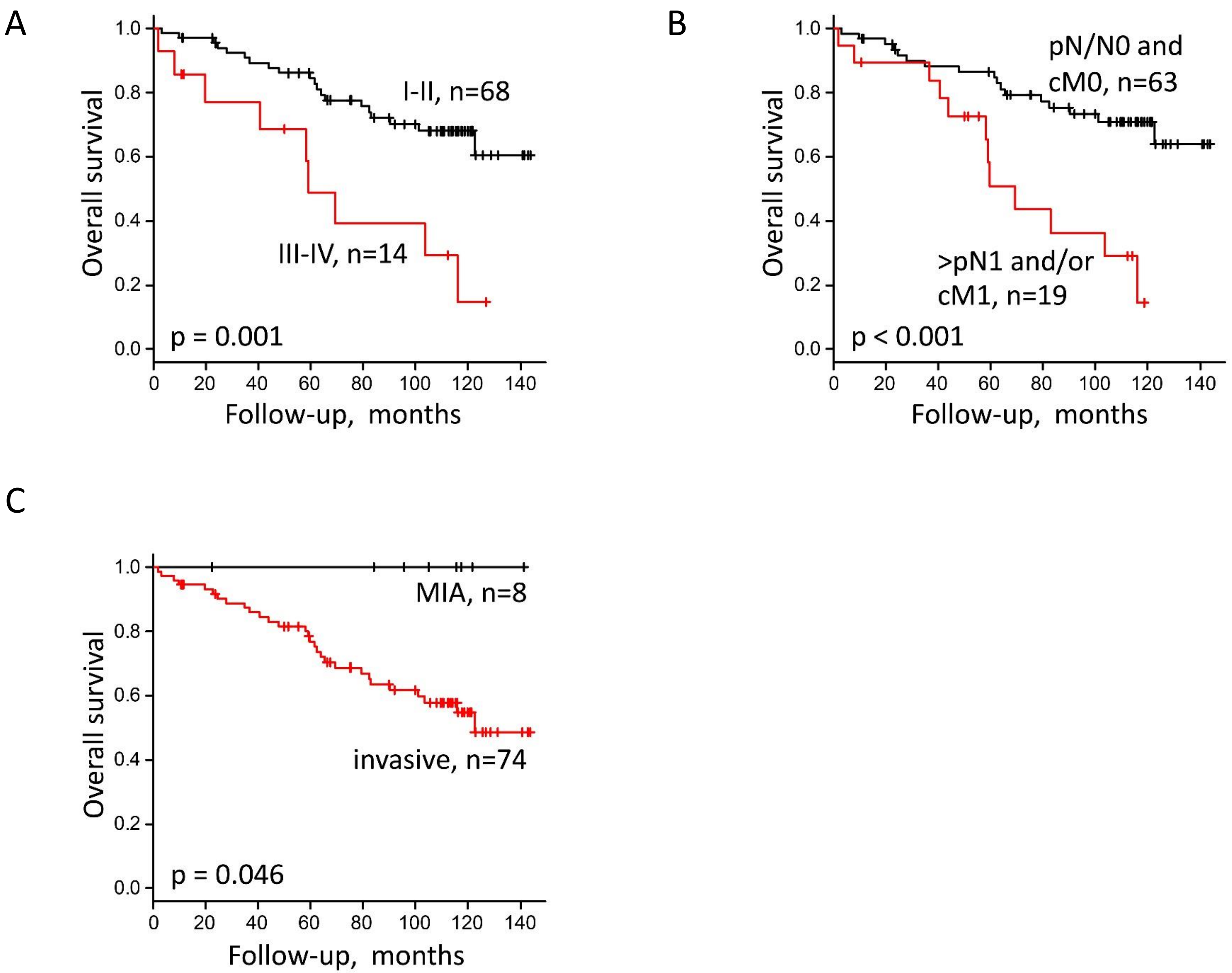
A
1.0
0.8
0.6
0.4
0.2
0.0
Overall survival
I-II, n=68
III-IV, n=14
p = 0.001
0
20
40
60
80
100
120
140
Follow-up, months
B
pN/N0 and
cM0, n=63
>pN1 and/or
cM1, n=19
p < 0.001
C
MIA, n=8
invasive, n=74
p = 0.046

Figure S5

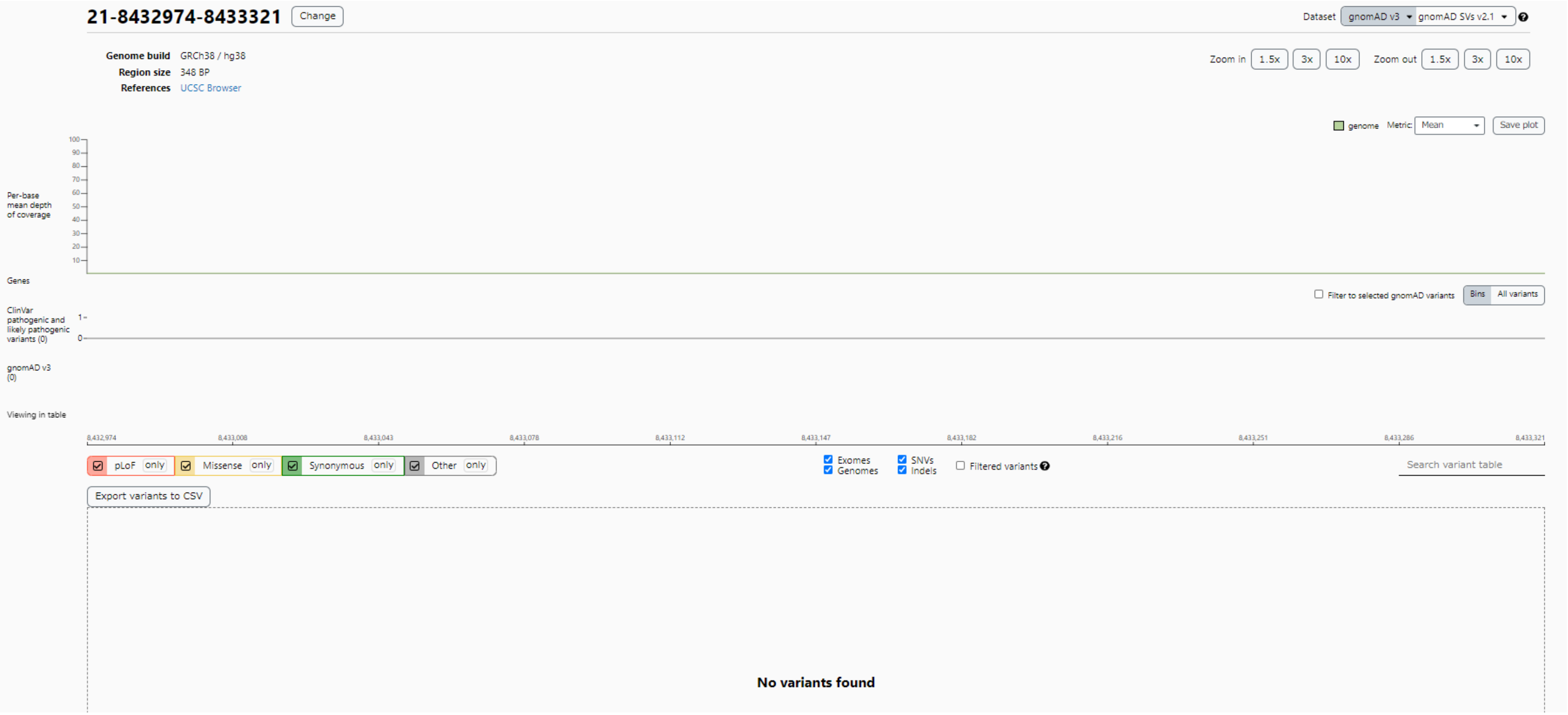
21-8432974-8433321
Change
Dataset gnomAD v3 gnomAD SVs v2.1
Genome build GRCh38 / hg38
Region size 348 BP
References UCSC Browser
Zoom in 1.5x 3x 10x Zoom out 1.5x 3x 10x
genome Metric: Mean Save plot
Per-base mean depth of coverage
Genes
Filter to selected gnomAD variants Bins All variants
ClinVar pathogenic and likely pathogenic variants (0)
gnomAD v3 (0)
Viewing in table
8,432,974
8,433,008
8,433,043
8,433,078
8,433,112
8,433,147
8,433,182
8,433,216
8,433,251
8,433,286
8,433,321
pLoF only Missense only Synonymous only Other only
Exomes Genomes
SNVs Indels
Filtered variants
Search variant table
Export variants to CSV
No variants found
