# Supplementary material for: Frequent Germline and Somatic Single Nucleotide Variants in the Promoter Region of the Ribosomal RNA Gene in Japanese Lung Adenocarcinoma Patients
Source: Cells. 2020 Nov 3;9(11):2409. doi: 10.3390/cells9112409 (PMC7692307; doi:10.3390/cells9112409)
Supplement: Supplementary file 1 [file cells-09-02409-s001.zip › Supplementary files/cells-942745 - Supplementary Tables S1-S3.docx]

**Table 1.** Primers used in this study.

| **Primer** | **Site** ^1^ | **AL592188** ^2^ | **Forward primer** | **Reverse primer** |
| --- | --- | --- | --- | --- |
| hrDNA  pro1 | -184 to +15 | 105240 to 105438, 149212 to 149410 | GTGTCCTGGGGTTGACCA | GGACAGCGTGTCAGCAATAA |
| hrDNA  pro2 | -282 to  -110 | 105142 to 105314, 149114 to 149286 | AAAATGCTTCCGGCTCCCG | ACACCTGTCCCCAAAAACGC |
| hrDNA  pro3 | -53 to +151 | 105371 to 105574, 149343 to 149546 | CGGGGGAGGTATATCTTTCGC | ACGCGCGAGAGAACAGCAG |
| hrDNA  18S | +5095 to +5388 | 110516 to 110807, 154488 to 154779 | ACTTCTTAGAGGGACAAGTGGC | AAACCATCCAATCGGTAGTAGC |
| hrDNA  28S | +12210 to +12506 | 117653 to 117949, 161625 to end | TACGAATACAGACCGTGAAAGC | TACTGAGCAGGATTACCATGGC |
| 47S/45S pre-rRNA |  |  | GAACGGTGGTGTGTCGTTC | GCGTCTCGTCTCGTCTCACT |
| 28S rRNA |  |  | TGTCGGCTCTTCCTATCATTGT | ACCCAGCTCACGTTCCCTATTA |
| Cyclophilin |  |  | TTCGTGCTCTGAGCACTGGAGA | GGACCCGTATGCTTTAGGATGAAG |

^1^ Site is relative to rRNA transcription start, according to GenBank U13369.; ^2^ Nucleotide position number is in reference to GenBank AL592188.

**Table 2.** The rDNA promoter SNVs (from the position -248 to +1) found in lung adenocarcinoma patients.

| **Patient** | **Histology** | **Sex** | **Age** | **Stage** | **Germline** | **Somatic** | **Months**  **post-surgery** | **Alive/Deceased** |
| --- | --- | --- | --- | --- | --- | --- | --- | --- |
| S1 | micropapillary | F | 66 | IIIA | -206 | -96 | 59.8 | Deceased |
| S2 | solid | M | 65 | IIIA | -206 |  | 19.8 | Deceased |
| S3 | MIA | M | 79 | IA1 | -206 |  | 117.4 | Alive |
| S4 | lepidic | M | 83 | IB | -206 |  | 11.0 | Alive |
| S5 | acinar | M | 62 | IIIB | -206 |  | 113.9 | Alive |
| S6 | papillary | M | 62 | IB | -206 |  | 109.6 | Alive |
| S7 | solid | M | 60 | IIB | -206 |  | 133.2 | Alive |
| S8 | IMA | M | 63 | IIB | -206 |  | 127.5 | Alive |
| S9 | papillary | M | 60 | IB | -204 |  | 114.1 | Alive |
| S10 | papillary | F | 58 | IIIA | -96 |  | 117.6 | Deceased |
| S11 | papillary | M | 70 | IA3 | -96 |  | 102.4 | Deceased |
| S12 | lepidic | F | 70 | IB | -96 |  | 112.5 | Alive |
| S13 | acinar | M | 68 | IV | -96 |  | 10.7 | Alive |
| S14 | papillary | F | 74 | IA | -96 |  | 68.6 | Alive |
| S15 | papillary | M | 59 | IA2 | -96 |  | 60.1 | Alive |
| S16 | IMA | F | 76 | IA2 | -96 |  | 107.3 | Alive |
| S17 | IMA | F | 62 | IA1 | -96 |  | 119.4 | Alive |
| S18 | MIA | F | 62 | IA1 |  | -96 | 97.0 | Alive |
| S19 | papillary (ASQ) | M | 59 | IIIA |  | -96 | 128.5 | Alive |
| S20 | MIA | M | 64 | IA1 |  | -72 | 106.6 | Alive |
| S21 | solid | M | 68 | IB |  | -72 | 122.5 | Alive |

rDNA, ribosomal RNA gene, ribosomal DNA; SNV, single nucleotide variant; MIA, minimally invasive adenocarcinoma; IMA, invasive mucinous adenocarcinoma; ASQ, adenosquamous carcinoma; M, male; F, female; Stage, UICC TNM stage.

**Table 3.** Survival analysis (log-rank test) for the rDNA promoter SNVs at each sites.

|  | **Recurrence-free survival** | | | | | | | | **Overall survival** | | |
| --- | --- | --- | --- | --- | --- | --- | --- | --- | --- | --- | --- |
|  | **Germline** | |  | **Somatic** | |  | **Total** | | **Germline** | **Somatic** | **Total** |
| SNVs | *n* | *P* |  | *n* | *P* |  | *n* | *P* | *P* | *P* | *P* |
| -206 | 9 | n.s. |  | 0 | - |  | 9 | n.s. | n.s. | - | n.s. |
| -96 | 8 | n.s. |  | 3 | n.s. |  | 11 | n.s. | n.s. | n.s. | n.s. |
| -72 | 0 | - |  | 3 | n.s. |  | 3 | n.s. | - | n.s. | n.s. |
| +49 | 2 | n.s. |  | 0 | - |  | 2 | n.s. | 0.001* | - | n.s. |
| +52 | 10 | n.s. |  | 2 | n.s. |  | 12 | n.s. | 0.035* | n.s. | n.s. |
| -248 to +1 | 20 | n.s. |  | 7 | n.s. |  | 25 | n.s. | n.s. | n.s. | n.s. |
| +1 to +100 | 12 | 0.030* |  | 2 | n.s. |  | 14 | n.s. | 0.004* | n.s. | 0.025* |
| Total (-248 to +100) | 29 | n.s. |  | 8 | n.s. |  | 35 | n.s. | n.s. | n.s. | n.s. |

rDNA, ribosomal RNA gene, ribosomal DNA; SNV, single nucleotide variant; n, number of the cases with each mutation; Total, Germline and/or somatic mutations; n.s., not significant. **P* < 0.05.
